# Intraocular manifestation of a diffuse large B-cell non-Hodgkin lymphoma of the pancreas

**DOI:** 10.1038/s41433-024-03259-y

**Published:** 2024-07-22

**Authors:** Colya N. Englisch, Tim Berger, Fidelis Flockerzi, Andrea Hasenfus, Berthold Seitz

**Affiliations:** 1 Department of Ophthalmology, Saarland University Medical Center, Homburg/Saar, Germany; 2 Department of Experimental Ophthalmology, Saarland University, Homburg/Saar, Germany; 3 Institute of Pathology, Saarland University Medical Center, Homburg/Saar, Germany

**Keywords:** Lymphoma, Lymphoma

**Fig. 1:**
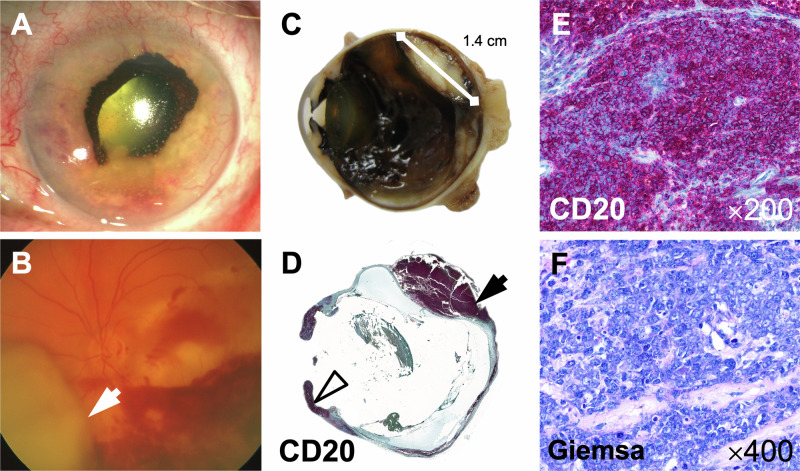
Clinical and histopathologic findings of a bulbar enucleate with intraocular manifestation of a diffuse large B-cell non-Hodgkin lymphoma of the pancreas. The anterior segment of the eye shows granulomatous corneal precipitates, fibrin exudate in the anterior chamber, and a massive rubeosis iridis with ectropion uveae (**A**). Funduscopic findings include a nasal, prominent, grayish mass (arrow) and marked retinal hemorrhages with whitish retinal infiltrates (**B**). The largest mass measured 1.4 × 1.2 × 0.5 cm and extended from anteronasal to posterotemporal (**C**). The histologic overview image (**D**, CD20-immunohistochemistry) shows extensive lymphoma manifestations in almost the complete retina and choroid, the ciliary body, and the iris (arrowhead). The lesion highlighted in (**C**) is marked with an arrow in (**D**). Higher magnifications display infiltrates of medium to large blastic tumor cells with loosened nuclear chromatin, prominent nucleoli, and mitotic figures (**E**: CD20-immunohistochemistry, **F**: Giemsa staining).

